# Comparison of Cardiorespiratory Fitness between Patients with Mitral Valve Prolapse and Healthy Peers: Findings from Serial Cardiopulmonary Exercise Testing

**DOI:** 10.3390/jcdd10040167

**Published:** 2023-04-13

**Authors:** Jin-Hui Chung, Yi-Ju Tsai, Ko-Long Lin, Ken-Pen Weng, Ming-Hsuan Huang, Guan-Bo Chen, Sheng-Hui Tuan

**Affiliations:** 1 Department of Physical Medicine and Rehabilitation, Kaohsiung Veterans General Hospital, Kaohsiung City 813414, Taiwan; 2 Department of Physical Therapy, Shu-Zen junior College of Medicine and Management, Kaohsiung City 82144, Taiwan; 3 Institute of Allied Health Sciences, College of Medicine, National Cheng Kung University, No.1, University Rd., Tainan City 70101, Taiwan; 4 Department of Physical Therapy, College of Medicine, National Cheng Kung University, Tainan 70101, Taiwan; 5 School of Medicine, National Yang Ming Chiao Tung University, Taipei 112304, Taiwan; 6 School of Medicine, College of Medicine, Kaohsiung Medical University, Kaohsiung 8073748, Taiwan; 7 Department of Post-Baccalaureate Medicine, National Sun Yat-Sen University, Kaohsiung 804315, Taiwan; 8 Congenital Structural Heart Disease Center, Department of Pediatrics, Kaohsiung Veterans General Hospital, Kaohsiung 813414, Taiwan; 9 Department of Internal Medicine, Kaohsiung Armed Forces General Hospital, Kaohsiung 802301, Taiwan; 10 Department of Rehabilitation Medicine, Cishan Hospital, Ministry of Health and Welfare, Kaohsiung 84247, Taiwan

**Keywords:** floppy mitral valve, valvular heart disease, aerobic fitness, exercise capacity, cardiopulmonary exercise testing, peak rate pressure product, early adolescence, late adolescence

## Abstract

Individuals with mitral valve prolapse (MVP) have exercise intolerance even without mitral valve regurgitation. Mitral valve degeneration may progress with aging. We aimed to evaluate the influence of MVP on the cardiopulmonary function (CPF) of individuals with MVP through serial follow-ups from early to late adolescence. Thirty patients with MVP receiving at least two cardiopulmonary exercise tests (CPETs) using a treadmill (MVP group) were retrospectively analyzed. Age-, sex-, and body mass index-matched healthy peers, who also had serial CPETs, were recruited as the control group. The average time from the first CPET to the last CPET was 4.28 and 4.06 years in the MVP and control groups, respectively. At the first CPET, the MVP group had a significantly lower peak rate pressure product (PRPP) than the control group (*p* = 0.022). At the final CEPT, the MVP group had lower peak metabolic equivalent (MET, *p* = 0.032) and PRPP (*p* = 0.031). Moreover, the MVP group had lower peak MET and PRPP as they aged, whereas healthy peers had higher peak MET (*p* = 0.034) and PRPP (*p* = 0.047) as they aged. Individuals with MVP had poorer CPF than healthy individuals as they develop from early to late adolescence. It is important for individuals with MVP to receive regular CPET follow-ups.

## 1. Introduction

Mitral valve prolapse (MVP), also known as floppy mitral valve syndrome, is a common valvular heart disease disorder that affects up to 3% of the general population [1], and the prevalence is approximately 3.36% in the overall Taiwanese population [2]. MVP represents the most common cause of primary mitral regurgitation (MR) in Western countries [3], and the severity of MVP can range from small or no leakage to severe MR. Although the Framingham Heart Study has shown that MVP is regarded as a benign condition with a low incidence of complications in the general population, 4% of MVP cases can be associated with significant MR [4]. Moreover, studies have uncovered several associated adverse events, including ischemic stroke [5], bacterial endocarditis [6], lethal arrhythmia [7], and sudden MVP-related cardiac death [4].

Given that the overall prognosis of MVP is benign and most individuals with MVP are asymptomatic, many patients with MVP do not seek medical help until complications occur [8]. Based on a large community-based study by Freed et al., the prevalence of adverse sequelae commonly associated with MVP was lower than that previously indicated by literature [9]; therefore, a renewed interest in stratifying the risk of MVP is advocated for because of recent new findings [8], including anatomo-pathological findings of MVP [10] and newly developed multimodality imaging of MVP [11]. Moreover, MVP is not observed in newborns, and its prevalence is approximately 0.3% in children and 0.6% in adolescents [12], which is much lower than that of the general population, suggesting that MVP is a progressive degenerative disease. Furthermore, pathophysiologic evidence shows that MVP is the primary myxomatous degeneration of one or both mitral valve leaflets. Over time, the patient may develop mitral annular dilatation, resulting in further cardiac systolic function worsening [12]. Therefore, individuals with MVP may need long-term and regular follow-ups for possible complications.

The degree of MR and ejection fraction is the major predictor of mortality in MVP [13]. Most methods used to evaluate the degree of MR and left ventricular function are implemented with patients at rest. However, as symptoms are frequently related to physical activity or exercise, there may be a poor correlation with indexes of the left ventricular function obtained at rest [14]. Cardiopulmonary exercise tests (CPETs) can obtain information about the integration among the cardiovascular, pulmonary, and musculoskeletal systems and the changes in the functional aerobic capacity due to pathological conditions during exercise [15]. CPET is considered the gold standard in assessing aerobic capacity and formulating function-based prognostic stratification among different nondiseased or diseased populations [16].

Studies suggest that individuals with MVP may have exercise intolerance [17]. They may present with symptoms that limit their exercise performance at school or work or may feel subjectively strained when performing physical activity. The underlying mechanisms of exercise intolerance in patients with MVP remain unclear. Autonomic dysfunction [18], a hyperadrenergic state [19], reduced left ventricular filling [20], catecholamine excess [20], or combinations thereof have been reported by studies. Nevertheless, the pathophysiologic basis underlying most of the associations remains controversial. Moreover, MVP symptoms are frequently disproportionate to objective findings through exhaustive clinical and laboratory investigation. Although several studies have observed that advanced MR impairs exercise tolerance independent of left ventricular ischemia and dysfunction [21,22], little is known regarding the influence of trivial or mild MR on exercise capacity.

Recently, our team observed that patients with MVP had similar exercise capacity (as indicated by peak metabolic equivalent [MET] measured during CPET); however, they had significantly lower peak rate pressure product (PRPP) values than healthy peers. The reduced PRPP indicates compromised coronary perfusion and subtle left ventricular function impairment in patients with MVP [23]. To our best knowledge, this is the first study in the literature that uses this method in patients with MVP. Moreover, since MVP results from mitral valve myxomatous degeneration and laxity, the MR of MVP patients may change over time. To date, little is known regarding exercise capacity, which is measured directly by CPET. Therefore, we aimed to observe the difference in physiologic responses to exercise testing between patients with MVP (without or with mild/moderate MR) and their healthy peers, from early to late adolescence, through serial follow-up.

## 2. Materials and Methods

### 2.1. Participant Characteristics

This was a retrospective cohort study, and the data were obtained from one medical center in Southern Taiwan (which has more than 20 years of experience in cardiopulmonary exercise testing and cardiopulmonary rehabilitation, performing more than 1500 cases of CPET for adults and around 500 cases of CPET for children per year). From July 2012 to June 2022, all children and adolescents aged 5–18 years referred from the Pediatric Cardiology Outpatient Clinic to the Department of Rehabilitation for CPET with the diagnosis of MVP were recruited. MVP diagnosis was made through a two-dimensional echocardiographic study by one well-experienced cardiologist (K.P.W.). MR severity was initially graded following consensus guidelines based on a four-point scale, as recommended by the American Society of Echocardiography, including mild (1+/4+), moderate (2+/4+), moderate to severe (3+/4+), and severe (4+/4+) [24]. All the recruited participants were required to meet the following additional inclusion criteria: participants who (A) underwent at least two symptom-limited treadmill exercise tests at an interval of more than 1 year, (B) completed transthoracic echocardiographic examination with preserved left ventricular ejection fraction (LVEF), and (C) underwent standard 12–lead electrocardiogram. Given that the aim of this study was to evaluate if there is any earlier change in the cardiopulmonary function in MVP patients without the development of advanced MR, MVP patients with co-existing advanced (moderate-to-severe and severe) MR were excluded. The other exclusion criteria included participants with (A) other congenital heart diseases (e.g., patent ductus arteriosus, ventricular septal defect, pulmonary artery stenosis, and atrial septal defect); (B) preexisting pulmonary disease; (C) significant arrhythmia; (D) significant coronary artery disease; and (E) missing data or incomplete exercise tests. To avoid the potential association between developmental aspects or body composition and exercise capacity [25,26], we recruited not only age- and sex-matched controls but also body mass index (BMI)-matched children and adolescents referred to the Pediatric Cardiology Outpatient Clinic in the same recruitment period for chest pain or dyspnea on exertion. Those in the control group needed to undergo CPET and have normal findings following echocardiography and a 12–lead electrocardiogram by cardiologists after medical records were reviewed by two experienced clinicians (G.B.C. and M.H.H.).

Although this was a retrospective study, we needed to recruit as many eligible participants as possible. We did a sample size estimation based on the statistical G*Power software (version 3.1.9.2, for Windows). Considering the study purpose, a two-tailed test with 0.8 effect size (high effect size as indicated in the study by Guimarães et al. [14], which showed that Cohen’s d was 0.87 for peak VO2 mL/kg/min), alpha of 0.05, and power of 0.80 with equally sized groups, was used [27] and yielded a sample size of 52, with 26 participants in each group to detect the effect.

All recruited participants completed transthoracic echocardiographic examination and standard 12–lead electrocardiogram at the Pediatric Cardiology Outpatient Clinic before their referral to the Rehabilitation Department. Within two weeks after the referral, the participants underwent body composition measurement, followed by a pulmonary function test and a symptom-limited treadmill exercise test. Patients’ characteristics and demographic variables, including age, sex, body weight and height, BMI, body fat, blood pressure, and pulse rate during rest, were recorded. The study was conducted following the principles outlined in the Helsinki Declaration and was approved by the Institutional Review Board of Kaohsiung Veterans General Hospital (number: VGHKS17-CT11-11). The study adhered to the STROBE checklist.

### 2.2. Cardiopulmonary Exercise Testing

To measure their exercise capacity, all participants underwent the symptom-limited exercise test, which was composed of a treadmill, a flow module, a gas analyzer, and an electrocardiographic monitor (Metamax 3B, Cortex Biophysik GmbH Co., Leipzig, Germany). The entire testing process was supervised by a physiatrist who had more than 20 years of experience in CPET (K.L.L.). We used the Bruce ramp protocol suggested by the American College of Sports Medicine during the entire CPET [28]. The exercise test was terminated when the participants demonstrated subjectively unbearable symptoms, when they could no longer continue, or when they attained maximal effort as indicated by the ACSM [28]. Oxygen consumption (VO2) and carbon dioxide production (VCO2) were measured using the breath-by-breath method during the exercise test. Furthermore, blood pressure (BP), HR, HR reserve (defined as peak HR minus baseline HR), and respiratory exchange ratio (RER, calculated as VCO2/VO2) were measured throughout the exercise test. Peak VO2 was the maximum oxygen uptake measured at peak exercise. Peak oxygen pulse was calculated as peak VO2 divided by peak HR. Peak oxygen pulse is a measure for stroke volume and peripheral oxygen extraction during exercise. Measured VO2 was divided by a constant 3.5 mL · kg^−1^·min^−1^ to derive METs. The anaerobic threshold (AT) was determined using the VE/VO2 and VE/VCO2 methods [29]. PRPP was defined as peak systolic BP multiplied by peak HR.

### 2.3. Pulmonary Function Test

All participants underwent pulmonary function tests with measurement of the forced vital capacity (FVC), forced expiratory volume in 1 s (FEV1), and maximal voluntary ventilation (MVV) at rest by spirometry. We divided the measured FVC by the predicted FVC (FVCP), the measured FEV1 by the predicted FEV1 (FEV1P), and the measured MVV by the predicted MVV (MVVP). We calculated the predicted values of the abovementioned parameters based on the spirometric reference equations for healthy children and adolescents in Taiwan [30].

### 2.4. Statistical Analysis

All analyses were performed using Statistical Package for the Social Sciences for Windows, version 19.0 (IBM Corp., Armonk, NY, USA). Continuous data were presented as means ± standard deviations, and categorical variables were presented as absolute numbers or percentages. Normality and homoscedasticity were examined before each analysis. The percentage of change of each CPET variable between the first and final testing (% of Δ CPET variable) was calculated as each final CPET variable minus each corresponding first CPET variable and subsequently divided by each corresponding first CPET variable. The comparisons of the basic characteristics, first CPET, final CPET, and the % of Δ CPET between the patients with MVP and the controls were made using the independent *t*-test for normally distributed variables and the Mann–Whitney U test for non-normally distributed variables. For the intragroup comparisons between the initial and final values for the exercise test variables of each group, the paired Student *t*-test and the Wilcoxon signed-rank test were used for normally and non-normally distributed variables, respectively. A *p*-value of ≤0.05 was considered statistically significant.

## 3. Results

Thirty-six patients met the inclusion criteria. Among them, two, one, and three patients had preexisting pulmonary disease, incomplete medical records, and incomplete results of exercise testing, respectively. Therefore, 30 patients with MVP were recruited for the final analysis (MVP group). Among them, 12 (40%), 14 (46.7%), and 4 (13.3%) patients had no MR, mild MR, and moderate MR, respectively. Thirty age-, sex-, and BMI-matched healthy peers were thus retrieved from our database as the control group.

### 3.1. Demographic Characteristics

The mean ages of the MVP and control groups were 13.28 ± 3.24 and 13.62 ± 2.65 years, respectively, at the time of receiving the first CPET (*p* = 0.675). The average time from the first CPET to the last CPET was 4.28 ± 2.55 and 4.06 ± 2.18 years in the MVP and control groups, respectively (*p* = 0.721). The demographic characteristics of the MVP and control groups are presented in Table 1. No statistically significant difference in age, sex, body height, body weight, BMI, resting SBP, resting DBP, and resting HR was noted between the MVP and control groups (Table 1).

### 3.2. Data of Pulmonary Function Tests

No statistically significant difference in all the routine examined parameters of spirometry (e.g., FVC, FVCP, FEV1, FEV1P, MVV, and MVVP) was observed between the MVP and control groups at both the first and final CPET (Table 2, upper rows). Moreover, no significant difference in % of Δ FVC, % of Δ FVCP, % of Δ FEV1, % of Δ FEV1P, % of Δ MVV, and % of Δ MVVP was observed between the two groups (Table 3, upper rows).

### 3.3. Data of CPET

The comparisons of CPET variables between the two groups are shown in Table 2 (lower rows). At the first CPET, no statistically significant differences were observed in the routine parameters measured during the CPET, including HR at AT (AT HR), MET at AT (AT MET), peak MET, peak HR, peak RER, peak diastolic and systolic BP, and peak oxygen pulse, except for PRPP, of which the MVP group presented with a lower value (27,048.00 ± 3488.07 vs. 29,905.05 ± 4081.12, *p* = 0.022) (Table 2, left columns).

At the final CPET, the MVP group had lower peak MET (8.48 ± 1.76 vs. 9.66 ± 1.99, *p* = 0.032) and PRPP than the control group (26,805.89 ± 3103.76 vs. 29,894.21 ± 6053.24, *p* = 0.031). No statistically significant differences were observed in the other routine parameters measured during the CPET (Table 2, left columns). Notably, the peak RER of the MVP and control groups were 1.18 ± 0.09 and 1.22 ± 0.14 (*p* = 0.188) in the first CPET and 1.17 ± 0.12 and 1.20 ± 0.14 (*p* = 0.451) in the last CPET, respectively, indicating that both the MVP and control groups could reach maximal effort during the CPET.

The intragroup comparisons of CPET variables at the first and last CEPTs of each group are presented in Table 2. No statistically significant differences were observed in all the routine parameters measured during the CPET of each group except that the peak oxygen pulse was higher in the last CPET than in the first CPET in both of the groups (Table 2, left columns). Peak oxygen pulse is a measure of peak oxygen consumed per peak heartbeat. Therefore, it’s reasonable to observe these results since peak oxygen consumption increases as ageing.

The change of CPET variables during the serial tests is demonstrated in Table 3. The average number of exercise tests was 2.21 ± 0.63 and 1.98 ± 0.67 (*p* = 0.176) per patient in the MVP and control groups, respectively. We observed that participants in the MVP group had lower peak MET and PRPP, whereas healthy peers had higher peak MET and PRPP as they develop into late adolescence. Statistically significant differences were observed in the % of Δ peak MET (−5.25% ± 17.25% vs. 5.56% ± 16.58%, *p* = 0.034) and % of Δ PRPP (−4.39% ± 13.44% vs. 9.19% ± 31.75%, *p* = 0.047) (Table 3, lower rows).

## 4. Discussion

To our knowledge, our study is the first to investigate exercise capacity through serial CPETs of patients with MVP. We observed that participants with MVP had comparable exercise capacity with their healthy peers in early adolescence, except that they had lower PRPP. However, as they develop into late adolescence, along with PRPP, patients with MVP had lower peak MET than their peers. Furthermore, we observed that peak MET and PRPP gradually decreased in the MVP group, whereas both variables increased in the control group.

Several studies and one recent meta-analysis have proven that adolescents with congenital heart disease have lower exercise capacity than matched healthy controls [31]. However, literature discussing the exercise intolerance of patients with MVP is limited. Centikaya et al. observed that children with MVP more frequently report arrhythmia and syncope than the normal population [32]. Other studies reported abnormal exercise tests by recognizing abnormal electrocardiography [33] or due to lower exercise duration [18] instead of directly comparing the VO2. None of these studies used the CPET results as indicators to compare precise exercise capability. Our study used the CPET data to perform comparisons and noted that early adolescents with MVP had similar peak MET though significantly lower PRPP than healthy peers. PRPP could be considered an accurate reflection of the myocardial oxygen demand and workload [34,35]. Santos et al. reported that myocardial oxygen uptake increases when eccentric left ventricular hypertrophy occurs, leading to coronary blood flow reserve exhaustion [36]. Moreover, we observed that among patients with Kawasaki disease, those without coronary artery involvement had higher PRPP than those with coronary artery dilation [37]. Therefore, lower PRPP may indicate compromised coronary perfusion and subtle left ventricular function impairment in patients with MVP.

Furthermore, our study demonstrated that along with PRPP, late adolescents with MVP had lower peak MET than healthy controls. Patients with MVP may develop MR as they age since there may be potential for mitral valve prolapse and loss of leaflet apposition [17]. It is well known that MR severity may progress, even to left ventricular overload and dysfunction when approaching its advanced stage in some patients with MVP [38,39]. Studies performing CPET have proven that similar to at-rest severe MR, exercise-induced changes in MR severity may limit the stroke volume adaptation during exercise and therefore contribute to exercise capacity limitation among patients with heart failure with reduced LVEF [40,41]. However, we only recruited patients with MVP with preserved LVEF; our study excluded moderate-to-severe and severe MVP patients, and there were only patients with mild (46.7%) and moderate MR (13.3%) in the MVP group, suggesting that younger patients with MVP could experience earlier changes as they age before the reduced LVEF can be detected. One study used cardiovascular magnetic resonance to compare patients with MVP with preserved ejection fraction and a control group. The authors reported that significant basal inferolateral hypertrophy, left ventricular dilatation, and exaggerated posterior annular displacement were noted, even in the absence of significant MR or overt MVP [42]. Some altered geometry and mechanics the mitral annulus as mentioned above may account for a repetitive mechanical stretch to the valve and left ventricular myocardium, progressively leading to myxomatous degeneration and arrhythmogenic myocardial scars in patients with MVP [43]. Moreover, patients with MVP may have increased energy demand due to repeated traction-associated higher myocardial work state, which accounts for the lower PRPP and peak MET we observed among the older adolescents in this study [44].

Although the natural history of MVP is benign, MVP is the main cause of surgical intervention for severe MR in developed countries [45]. Chronic MR may cause pulmonary hypertension with subsequent heart failure and an increased risk of arrhythmias [46]. Patients with MVP also have an increased predisposition to arrhythmogenic sudden cardiac death [44]. Therefore, it is important to detect ventricular function abnormalities among patients with MVP early; we assumed that PRPP could be a potential indicator. However, further prospective studies regarding the association between PRPP and left-ventricular-related parameters are warranted.

Given the results of our study, we encourage all children and adolescents with MVP to engage in exercise. According to the consensus by the American Heart Association and American College of Cardiology, physically active individuals with MVP who have mild-to-moderate MR are encouraged to engage in any type of competitive sports; however, those with left ventricular systolic dysfunction, arrhythmias on Holter recording, or a family history of SCD can only participate in low-intensity competitive sports [47]. Exercise capacity quantification of patients with MVP using CPET is also important. During the CPET evaluation, the self-efficacy of patients with MVP is established by performing comparable physical fitness. Furthermore, physicians could distinguish exercise limitation due to anxiety alone from organic disease and prescribe a tailor-made exercise recommendation.

Our study had some limitations. First, this was a retrospective study. There may be variation in follow-up timing; however, all our recruited participants received CPET within days following complete echocardiography. Second, we only recruited participants from one medical center in Southern Taiwan; therefore, the results may not be applicable to the general population, and a larger cross-national study is needed for further evaluation. Third, although the final count of analyzed participants was more than the estimated minimum sample size, our sample size remained small with a retrospective nature. Fourth, those who had moderate-to-severe and severe MR were excluded from our study. Patients with MVP with prominent symptoms may directly seek surgical help instead of running through a detailed examination, which contributed to referral bias. Lastly, the participants in our study were referred by cardiovascular outpatient clinics and presented with variable symptoms, which may contribute to selection bias. Future prospective and cross-national studies using cardiac magnetic resonance findings or echocardiographic measurement for better prolapse severity quantification with a larger sample size are warranted.

## 5. Conclusions

Early adolescents with MVP had exercise capacities comparable to their healthy peers, except for PRPP. However, as they aged, the peak exercise load capacity of late adolescents was significantly lower than that of controls. This discrepancy in the CEPT between early and late adolescents may be because of altered geometry and mechanics resulting from mitral valve degeneration. More investigations are needed to further understand the complex interaction between anatomical, pathophysiological, and hemodynamic aspects in patients with MVP. Moreover, it is important for adolescents with MVP to undergo CPET and engage in exercise.

## Figures and Tables

**Table 1:** Demographic characteristics of patients with mitral valve prolapse and control group.

	MVP (*n* = 30)	Control (*n* = 30)	*p* Value ^a^
Age at first CPET(y)	13.28 ± 3.24	13.62 ± 2.65	0.675
Height (cm)	153.52 ± 11.72	158.38 ± 11.06	0.121
Weight (kg)	49.89 ± 14.57	53.09 ± 10.11	0.353
BMI (kg/m^2^)	20.88 ± 4.66	21.08 ± 3.14	0.853
Resting HR (bpm)	87.72 ± 15.25	86.38 ± 11.74	0.719
Resting SBP (mmHg)	114.79 ± 12.31	114.86 ± 18.44	0.988
Resting DBP (mmHg)	67.93 ± 7.27	64.64 ± 7.70	0.125
Time from first to last CPET (y)	4.28 ± 2.55	4.06 ± 2.18	0.721

Data are the mean ± standard deviation. MVP, mitral valve prolapse; BMI, body mass index; HR, heart rate; SBP, systolic blood pressure; DBP, diastolic blood pressure. ^a^ Refers to the *p* value of independent *t*-test (variables with normal distribution) or Mann–Whitney U-test (variables with non-normal distribution) between the two subgroups.

**Table 2:** Findings of the initial cardiopulmonary exercise testing among patients with mitral valve prolapse.

	First CPET			Last CPET			Comparisons between the 1st and the Last CPET	
	MVP (*n* = 30)	Control (*n* = 30)	*p* Value ^a^	MVP (*n* = 30)	Control (n = 30)	*p* Value ^a^	MVP *p* Value ^b^	Control *p* Value ^b^
FVC (L)	2.93 ± 0.57	3.03 ± 1.17	0.653	3.12 ± 0.48	3.27 ± 1.07	0.587	0.168	0.192
FVCP (%)	99.75 ± 21.23	98.15 ± 21.45	0.791	90.95 ± 12.65	95.44 ± 21.32	0.432	0.081	0.434
FEV1 (L)	2.38 ± 0.61	2.70 ± 11.12	0.212	2.72 ± 0.38	2.93 ± 1.09	0.431	0.093	0.233
FEV1P (%)	100.03 ± 20.70	101.09 ± 24.55	0.867	92.66 ± 11.58	94.66 ± 29.83	0.734	0.100	0.207
FEV1/FVC (%)	88.75 ± 80.5	88.96 ± 6.83	0.920	87.05 ± 5.36	88.81 ± 9.15	0.475	0.069	0.332
MVV (L)	67.60 ± 22.30	79.05 ± 35.00	0.136	74.75 ± 13.64	84.10 ± 25.78	0.188	0.140	0.245
MVVP (%)	100.75 ± 50.26	103.63 ± 57.82	0.834	81.80 ± 24.94	97.66 ± 54.99	0.156	0.069	0.684
AT HR (bpm)	146.72 ± 12.07	140.35 ± 13.05	0.065	141.33 ± 11.98	143.55 ± 12.50	0.485	0.146	0.336
AT MET	6.29 ± 1.43	6.25 ± 0.77	0.898	6.87 ± 5.46	6.92 ± 1.29	0.968	0.299	0.142
peak MET	9.11 ± 2.27	9.29 ± 1.78	0.744	8.48 ± 1.76	9.66 ± 1.99	0.032 *	0.235	0.451
peak HR (bpm)	179.48 ± 9.41	177.81 ± 11.01	0.546	175.77 ± 12.12	177.91 ± 7.94	0.422	0.191	0.973
peak RER	1.18 ± 0.09	1.22 ± 0.14	0.201	1.17 ± 0.12	1.20 ± 0.14	0.451	0.717	0.246
peak SBP (mmHg)	161.82 ± 21.76	155.96 ± 28.78	0.400	158.88 ± 20.60	164.96 ± 35.36	0.475	0.318	0.304
peak DBP (mmHg)	81.32 ± 20.07	76.96 ± 17.55	0.401	78.38 ± 15.34	80.65 ± 18.95	0.612	0.526	0.437
PRPP	27048.00 ± 3488.07	29905.05 ± 4081.12	0.022 *	26805.89 ± 3103.76	29894.21 ± 6053.24	0.031 *	0.777	0.367
peak oxygen pulse (mL/beat)	8.71 ± 2.82	9.68 ± 2.45	0.182	9.95 ± 2.16	10.92 ± 2.49	0.131	0.007 *	0.004 *

Data are the mean ± standard deviation; MVP, mitral valve prolapse; FVC, functional vital capacity; FVCP, percentage of predicted forced vital capacity; FEV1, forced expiratory volume at 1 min; FEV1P, percentage of predicted forced expiratory volume at 1 min; MVV, maximal voluntary ventilation; MVVP, percentage of predicted maximal voluntary ventilation; MET, metabolic equivalent; AT MET, MET at the point of anaerobic threshold; peak MET, largest MET during whole exercise testing; peak VO2, peak oxygen consumption; % of peak predicted, percentage of predicted peak VO2; HR, heart rate; peak RER, largest respiratory exchange ratio during whole exercise testing; SBP, systolic blood pressure; DBP, diastolic blood pressure; PRPP, peak rate pressure product. ^a^ Refers to the *p* value of independent *t*-test (variables with normal distribution) or Mann–Whitney U-test (variables with non-normal distribution) between the two subgroups. ^b^ Refers to the *p* value of paired *t*-test (variables with normal distribution) or Wilcoxon signed-rank test (variables with non-normal distribution) of each group. * *p* value < 0.05.

**Table 3:** Comparison of the change of variables of the last from the first cardiopulmonary exercise testing between the mitral valve prolapse group and the control group.

Difference (%) between the First and the Last CPET			
	MVP (*n* = 30)	Control (*n* = 30)	*p* Value ^a^
FVC (L)	0.12 ± 0.21	0.21 ± 0.41	0.409
FVCP (%)	−0.06 ± 0.18	−0.22 ± 0.20	0.565
FEV1 (L)	0.13 ± 0.32	0.23 ± 0.51	0.472
FEV1P (%)	−0.08 ± 0.21	−0.60 ± 0.25	0.832
FEV1/FVC (%)	−0.03 ± 0.06	−0.02 ± 0.09	0.764
MVV (L)	0.36 ± 0.54	0.29 ± 0.55	0.738
MVVP (%)	−0.03 ± 0.63	−0.17 ± 0.52	0.483
AT HR (bpm)	−0.04 ± 0.10	0.05 ± 0.10	0.002
AT MET	−0.03 ± 0.23	0.07 ± 0.22	0.127
peak MET	−5.25 ± 17.25	5.56 ± 16.58	0.034 *
peak HR (bpm)	−0.03 ± 0.07	0.00 ± 0.07	0.134
peak RER	0.00 ± 0.13	−0.02 ± 0.12	0.597
peak SBP (mmHg)	−0.02 ± 0.14	0.09 ± 0.28	0.084
peak DBP (mmHg)	−0.27 ± 0.27	0.15 ± 0.28	0.025
PRPP	−4.39 ± 13.44	9.19 ± 31.75	0.047 *
peak oxygen pulse (mL/beat)	22.14 ± 38.65	15.54 ± 25.13	0.462

Data are the mean ± standard deviation. MVP, mitral valve prolapse; FVC, functional vital capacity; FVCP, percentage of predicted forced vital capacity; FEV1, forced expiratory volume at 1 min; FEV1P, percentage of predicted forced expiratory volume at 1 min; MVV, maximal voluntary ventilation; MVVP, percentage of predicted maximal voluntary ventilation; MET, metabolic equivalent; AT MET, MET at the point of anaerobic threshold; peak MET, largest MET during whole exercise testing; peak VO2, peak oxygen consumption; % of peak predicted, percentage of predicted peak VO2; HR, heart rate; peak RER, largest respiratory exchange ratio during whole exercise testing; SBP, systolic blood pressure; DBP, diastolic blood pressure; PRPP, peak rate pressure product. ^a^ Refers to the *p* value of independent *t*-test (variables with normal distribution) or Mann–Whitney U-test (variables with non-normal distribution) between the two subgroups. * *p* value < 0.05

## Data Availability

Data sharing statement: Individual participant data that underlie the results reported in this article might be shared after deidentification. Proposals should be directed to Gabbrile@vghks.gov.tw for application.
